# Supplementary material for: N-terminal domain on dystroglycan enables LARGE1 to extend matriglycan on α-dystroglycan and prevents muscular dystrophy
Source: eLife. 2023 Feb 1;12:e82811. doi: 10.7554/eLife.82811 (PMC9917425; doi:10.7554/eLife.82811)
Supplement: Figure 2—source data 1. [file elife-82811-fig2-data1.zip › Figure 2C-source data 1/Figure 2C_8-22-22_data source.docx]

**
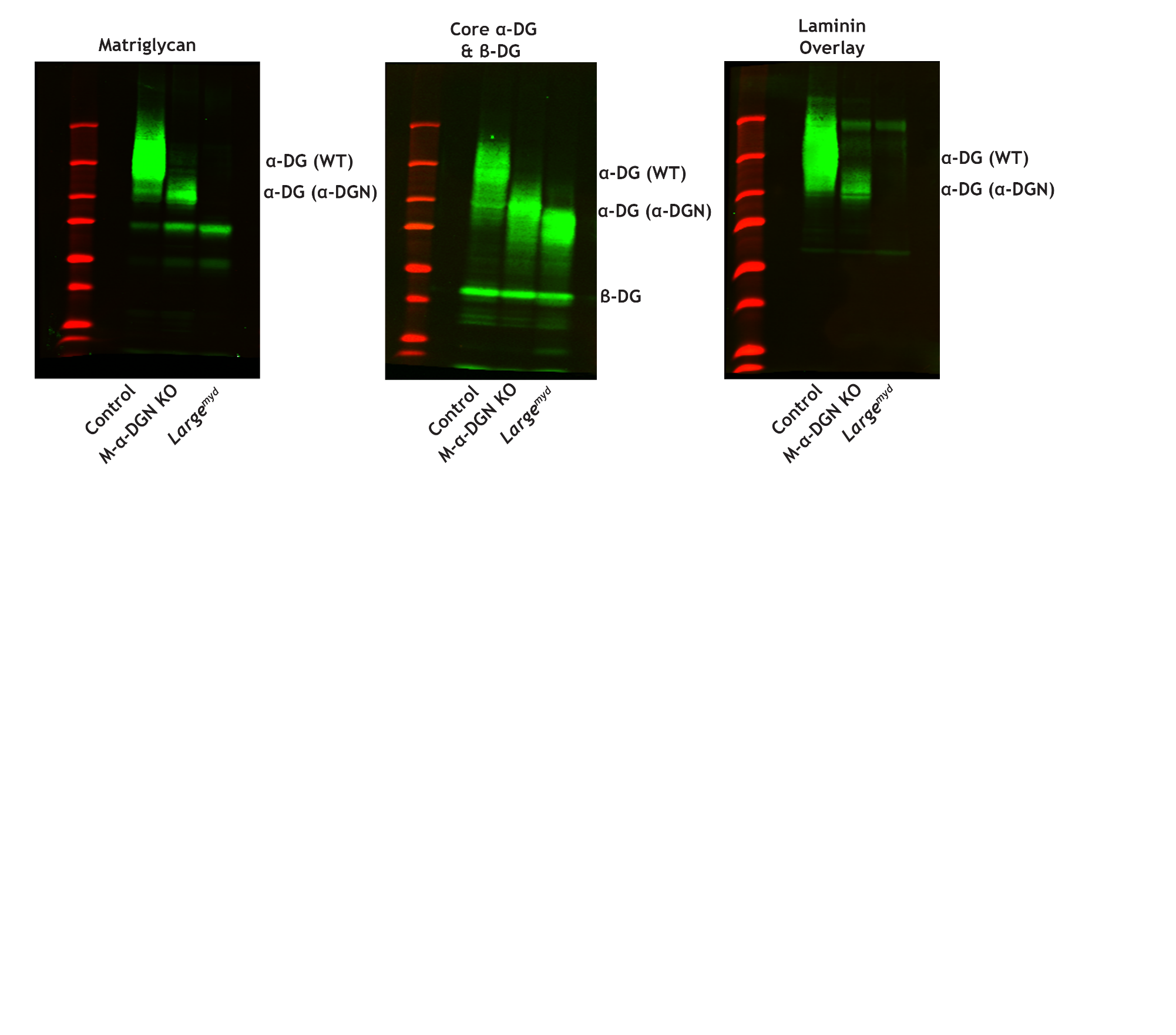
**

**Figure 2. Characterization of mice with a muscle-specific loss of α-DGN.** **(C)** Immunoblot analysis of skeletal muscle from control, M-α-DGN KO, and *Large^myd^* mice. Glycoproteins were enriched using wheat-germ agglutinin (WGA)-agarose with 10 mM EDTA. Immunoblotting was performed to detect matriglycan (IIIH11), core α-DG, β-DG (AF6868), and laminin overlay. α-DG in WT control muscle (α-DG (WT)) and α-DG in α-DGN-deficient muscle (α-DG (Δα-DGN)) are indicated on the right. Molecular weight standards in kilodaltons (kDa) are shown on the left. Molecular weight standards in kilodaltons (kDa) are shown on the left (250, 150, 100, 75, 50, 37, 25, and 20).
