# Supplementary material for: N-terminal domain on dystroglycan enables LARGE1 to extend matriglycan on α-dystroglycan and prevents muscular dystrophy
Source: eLife. 2023 Feb 1;12:e82811. doi: 10.7554/eLife.82811 (PMC9917425; doi:10.7554/eLife.82811)
Supplement: Figure 2—figure supplement 1—source data 1. [file elife-82811-fig2-figsupp1-data1.zip › Figure 2-figure supplement 1-source data 1/Figure 2-Supp1_1-18-23_data source.docx]

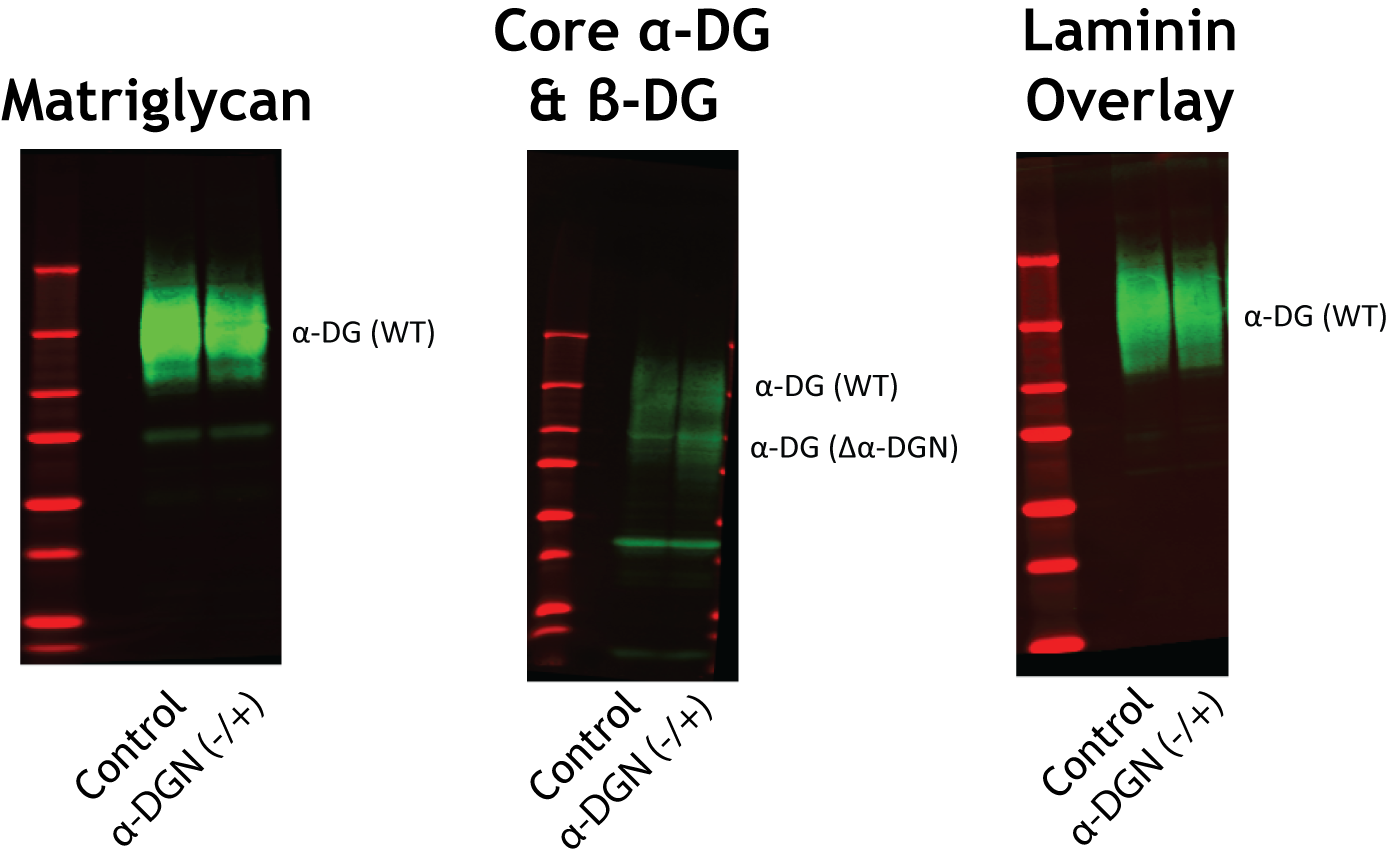


**Figure 2-figure supplement 1. Mice heterozygous (+/-) for a constitutive deletion of α-DGN have two different sizes of α-DG.** Immunoblot analysis of skeletal muscle from littermate controls or mice that are heterozygous for the α-DGN KO allele (α-DGN (-/+)). Glycoproteins were enriched from the quadriceps skeletal muscles of mice using WGA-agarose with 10 mM EDTA. Immunoblotting was performed to detect matriglycan (IIIH11), core α-DG and β-DG (AF6868), and laminin overlay. α-DG in WT control muscle (α-DG(WT)) and α-DG in α-DGN-deficient muscle (α-DG(Δα-DGN)) are indicated on the right. Molecular weight standards in kilodaltons (kDa) are shown on the left. Molecular weight standards in kilodaltons (kDa) are shown on the left (250, 150, 100, 75, 50, 37, 25, and 20).
