# Supplementary material for: N-terminal domain on dystroglycan enables LARGE1 to extend matriglycan on α-dystroglycan and prevents muscular dystrophy
Source: eLife. 2023 Feb 1;12:e82811. doi: 10.7554/eLife.82811 (PMC9917425; doi:10.7554/eLife.82811)
Supplement: Figure 2—figure supplement 2—source data 1. [file elife-82811-fig2-figsupp2-data1.zip › Figure 2-figure supplement 2-source data 1/Figure 2 - Supplement 2.docx]

**
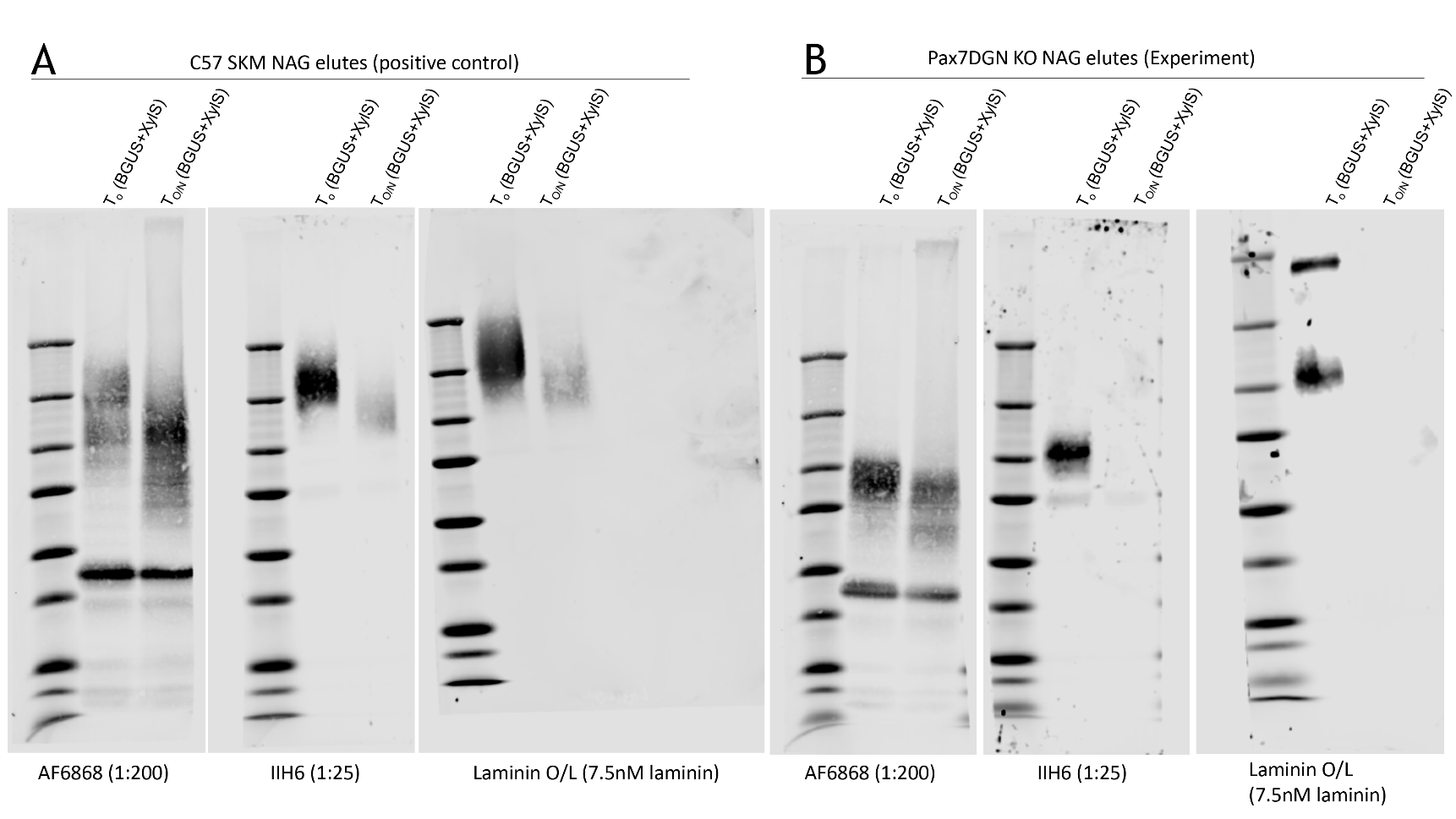
**

**Figure 2-figure supplement 2.** **The short 100-120 kDa band in M-α-DGN KO muscle is matriglycan.** **(A)** Immunoblot analysis of total skeletal muscle from control mice after digestion with enzymes β-glucuronidase and α-xylosidase. Glycoproteins were enriched using wheat-germ agglutinin (WGA)-agarose with 10 mM EDTA and incubated overnight with β-glucuronidase (BGUS) and α-xylosidase (XyIS). Immunoblotting was performed to detect matriglycan (IIH6), core α-DG and β-DG (AF6868), and laminin overlay before (T_o_) and after overnight digestion (T_O/N_). **(B)** Immunoblot analysis of M-α-DGN KO total skeletal muscle after digestion with enzymes BGUS and XyIS. Glycoproteins were enriched using wheat-germ agglutinin (WGA)-agarose with 10 mM EDTA and incubated overnight with BGUS and XyIS. Immunoblotting was performed to detect matriglycan (IIH6), core α-DG and β-DG (AF6868), and laminin overlay before (T_o_) and after digestion (T_O/N_). Molecular weight standards in kilodaltons (kDa) are shown on the left (250, 150, 100, 75, 50, 37, 25, 20, and 15).
