# Supplementary figures and images for: N-terminal domain on dystroglycan enables LARGE1 to extend matriglycan on α-dystroglycan and prevents muscular dystrophy
Source: eLife. 2023 Feb 1;12:e82811. doi: 10.7554/eLife.82811 (PMC9917425; doi:10.7554/eLife.82811)

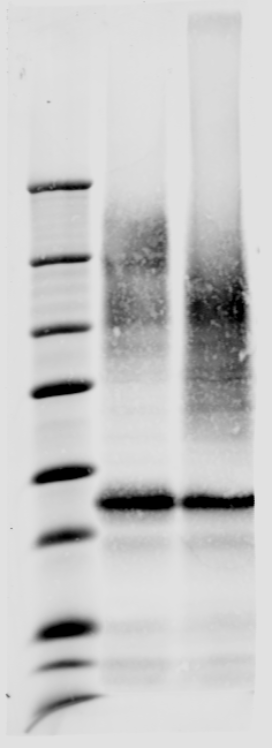

Supplement: Figure 2—figure supplement 2—source data 1. [file elife-82811-fig2-figsupp2-data1.zip › Figure 2-figure supplement 2-source data 1/C57 AF6868.tif]

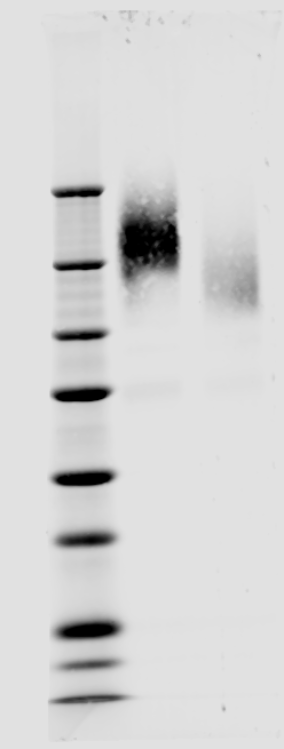

Supplement: Figure 2—figure supplement 2—source data 1. [file elife-82811-fig2-figsupp2-data1.zip › Figure 2-figure supplement 2-source data 1/C57 IIH6.tif]

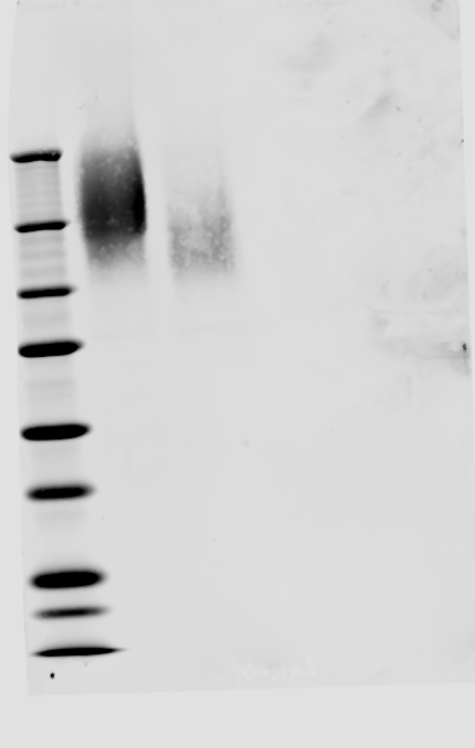

Supplement: Figure 2—figure supplement 2—source data 1. [file elife-82811-fig2-figsupp2-data1.zip › Figure 2-figure supplement 2-source data 1/C57 Lam OL.tif]

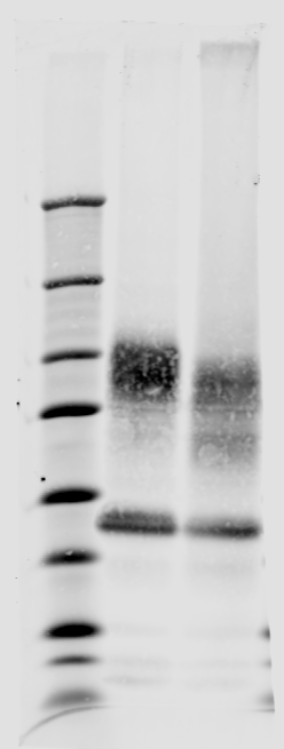

Supplement: Figure 2—figure supplement 2—source data 1. [file elife-82811-fig2-figsupp2-data1.zip › Figure 2-figure supplement 2-source data 1/Pax 7 DGN KO AF6868.tif]

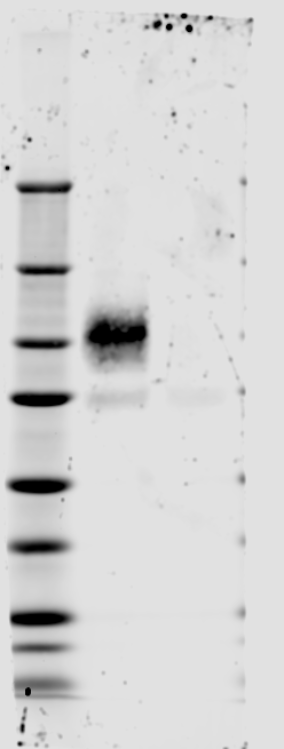

Supplement: Figure 2—figure supplement 2—source data 1. [file elife-82811-fig2-figsupp2-data1.zip › Figure 2-figure supplement 2-source data 1/Pax7DGN KO IIH6.tif]

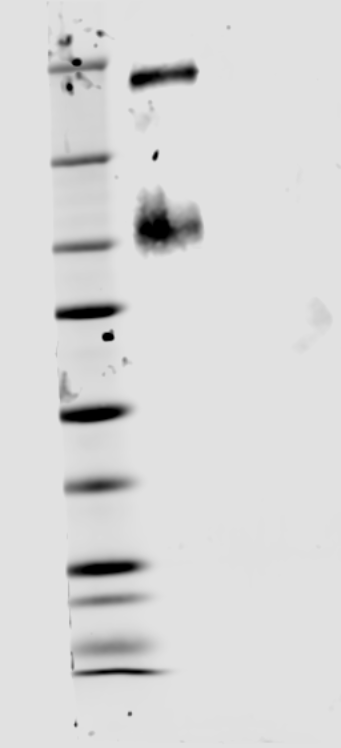

Supplement: Figure 2—figure supplement 2—source data 1. [file elife-82811-fig2-figsupp2-data1.zip › Figure 2-figure supplement 2-source data 1/Pax7DGN KO OL.tif]
