# Supplementary material for: N-terminal domain on dystroglycan enables LARGE1 to extend matriglycan on α-dystroglycan and prevents muscular dystrophy
Source: eLife. 2023 Feb 1;12:e82811. doi: 10.7554/eLife.82811 (PMC9917425; doi:10.7554/eLife.82811)
Supplement: Figure 5—source data 1. [file elife-82811-fig5-data1.zip › Figure 5C-source data 1/Figure 5C_1-18-23_red and green.docx]

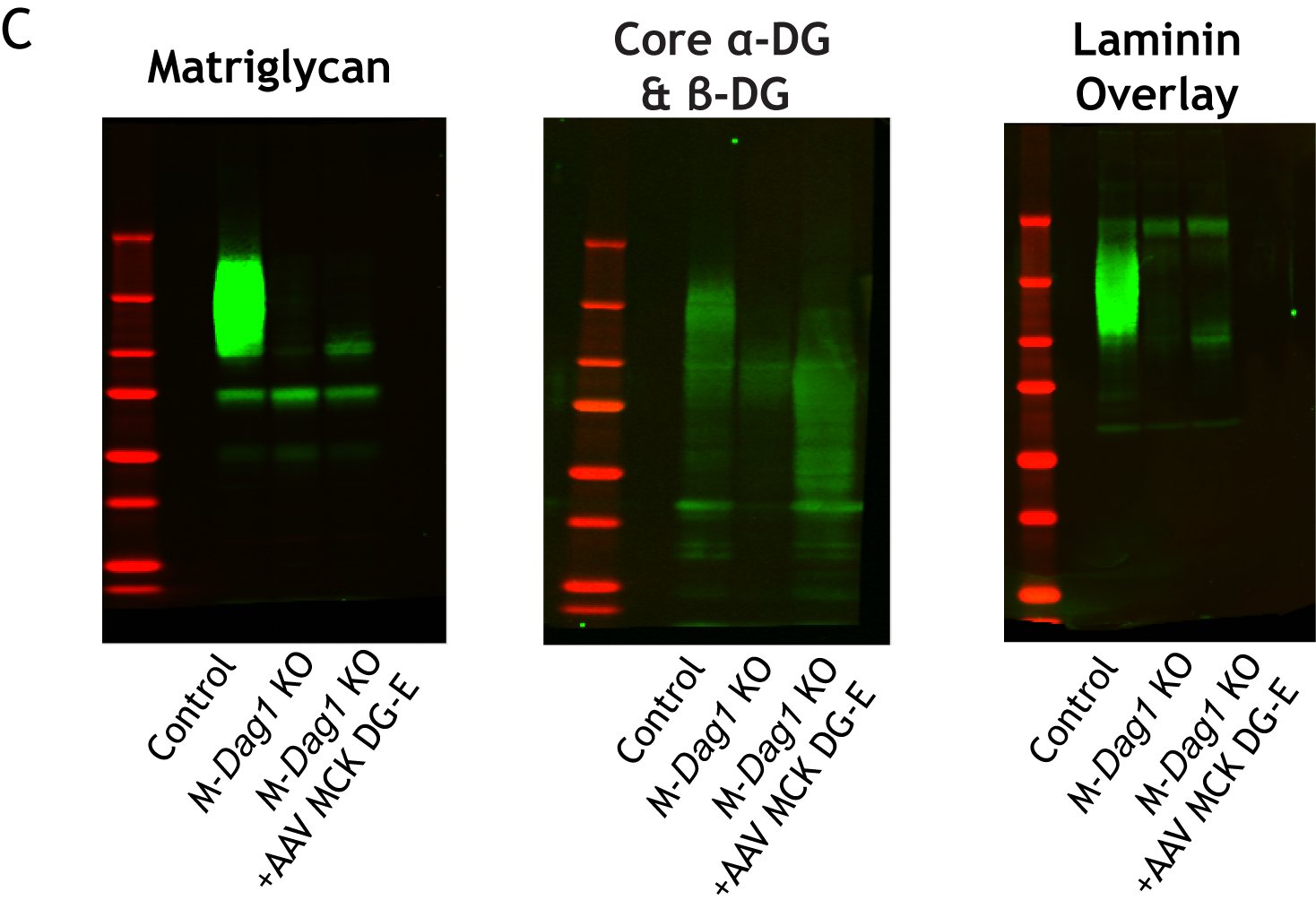


**Figure 5. Exogenous α-DGN-deficient DG also produces short matriglycan like M-*Dag1* KO muscle.** (**C**) Immunoblot analysis of skeletal muscle obtained from littermate controls (control), M-*Dag1* KO mice or M-*Dag1* KO mice injected with AAV-MCK DG-E. Glycoproteins were enriched from skeletal muscles using WGA-agarose. Immunoblotting was performed to detect matriglycan (IIIH11), core α-DG and β-DG (AF6868), and laminin (overlay). Molecular weight standards in kilodaltons (kDa) are shown on the left (250, 150, 100, 75, 50, 37, 25, and 20).
