# Supplementary material for: N-terminal domain on dystroglycan enables LARGE1 to extend matriglycan on α-dystroglycan and prevents muscular dystrophy
Source: eLife. 2023 Feb 1;12:e82811. doi: 10.7554/eLife.82811 (PMC9917425; doi:10.7554/eLife.82811)
Supplement: Figure 5—figure supplement 1—source data 1. [file elife-82811-fig5-figsupp1-data1.zip › Figure 5-figure supplement 1B-source data 1/Figure 5-Supp 1B_8-23-22_red and green_v2.docx]

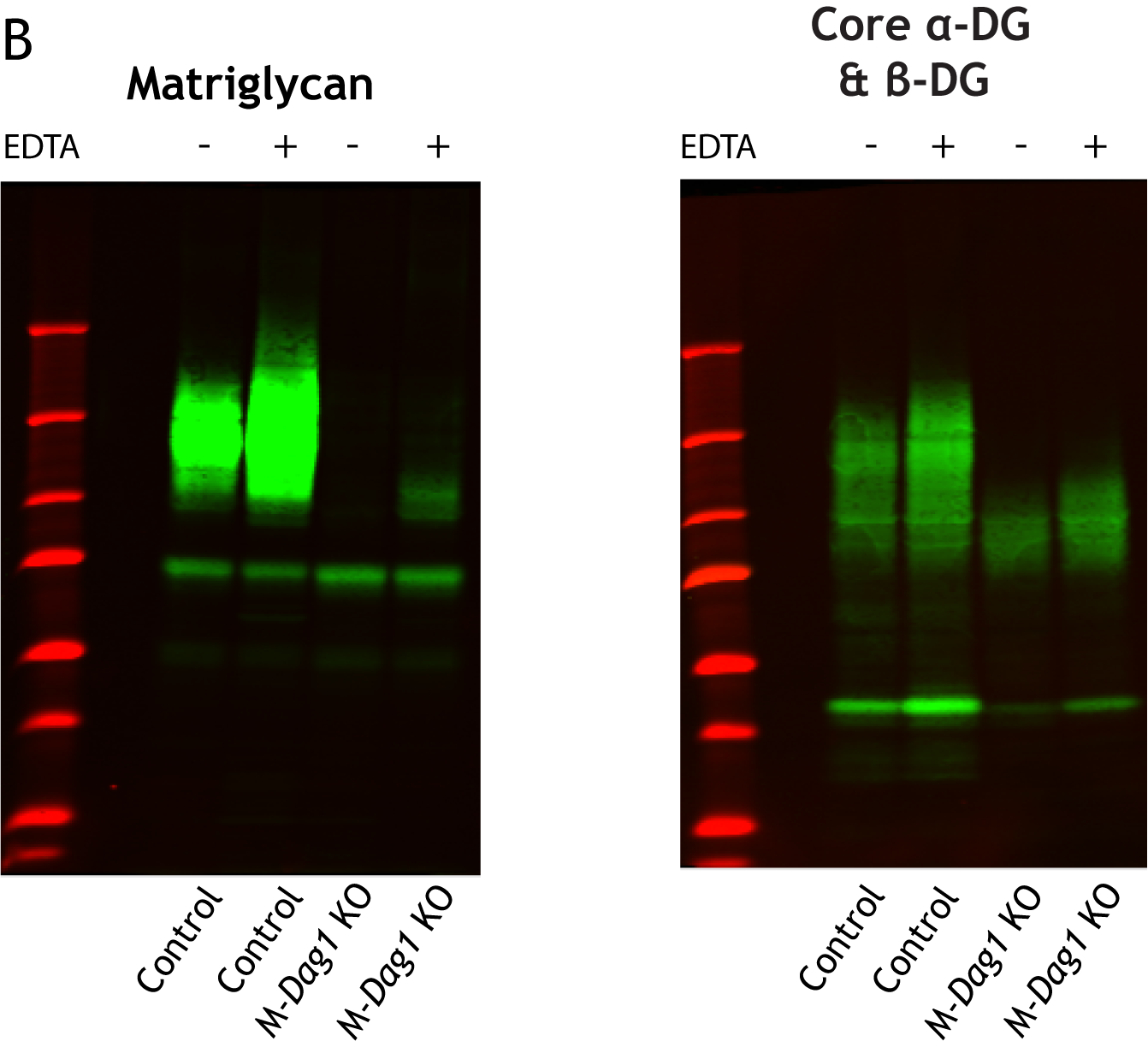


**Figure 5-figure supplement 1. Characteristics of M*-Dag1* KO (*Pax7^cre^; Dag1^flox/flox^)* mice. (B)** Immunoblot analysis of skeletal muscle from control and M-*Dag1* KO mice. Glycoproteins were enriched from skeletal muscles using WGA-agarose with (+) and without (-) 10 mM EDTA. Immunoblotting was performed to detect matriglycan (IIIH11) and core α-DG and β-DG (AF6868). Molecular weight standards in kilodaltons (kDa) are shown on the left (250, 150, 100, 75, 50, 37, 25, and 20).
