# Supplementary material for: N-terminal domain on dystroglycan enables LARGE1 to extend matriglycan on α-dystroglycan and prevents muscular dystrophy
Source: eLife. 2023 Feb 1;12:e82811. doi: 10.7554/eLife.82811 (PMC9917425; doi:10.7554/eLife.82811)
Supplement: Figure 5—figure supplement 2—source data 1. [file elife-82811-fig5-figsupp2-data1.zip › Figure 5-figure supplement 2-source data 1/Figure 5-Supp 2_1-17-23_source data file.docx]

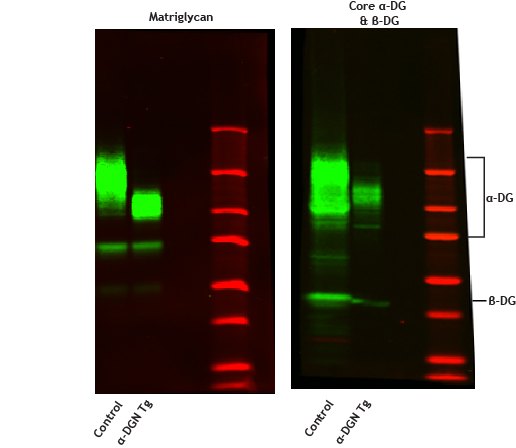


**Figure 5-figure supplement 2.** **Excess free α-DGN interferes with the LARGE1 elongation of matriglycan on α-DG.** Immunoblot analysis of skeletal muscle from control and α-DGN Tg mice. Glycoproteins were enriched using WGA-agarose. Immunoblotting was performed to detect matriglycan (IIH6), core α-DG, and β-DG (AF6868). Molecular weight standards in kilodaltons (kDa) are shown on the left (250, 150, 100, 75, 50, 37, 25, and 20).
