# Supplementary material for: N-terminal domain on dystroglycan enables LARGE1 to extend matriglycan on α-dystroglycan and prevents muscular dystrophy
Source: eLife. 2023 Feb 1;12:e82811. doi: 10.7554/eLife.82811 (PMC9917425; doi:10.7554/eLife.82811)
Supplement: Figure 6—source data 1. [file elife-82811-fig6-data1.zip › Figure 6B-source data 1/Figure 6B_1-18-23_red and green.docx]

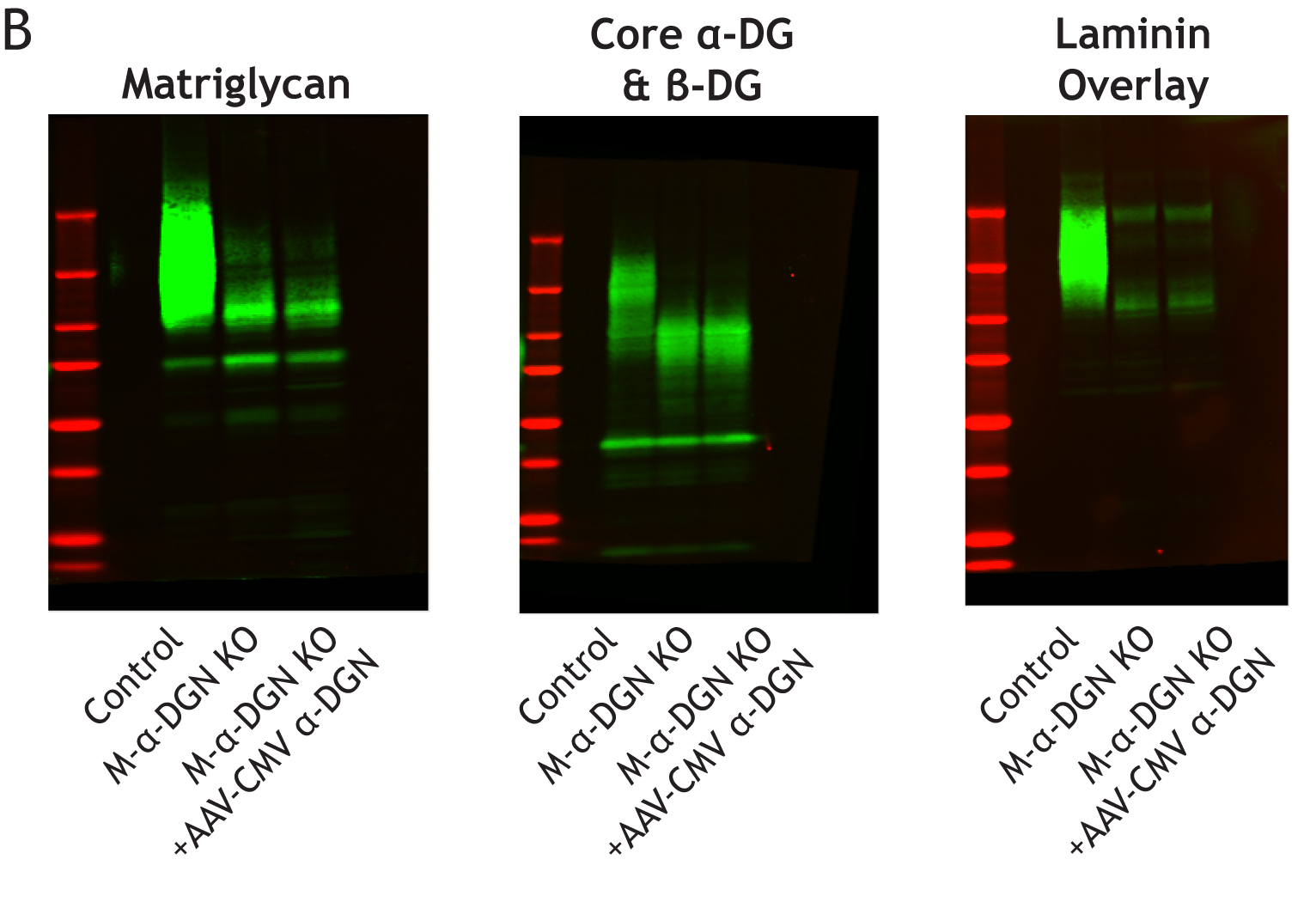


**Figure 6. Expression of α-DGN in M-α-DGN KO mice does not rescue matriglycan elongation. (B)** Immunoblot analysis of skeletal muscle obtained from littermate controls or M-α-DGN KO mice and M-α-DGN KO mice injected with AAV-CMV α-DGN (M-α-DGN KO+AAV-CMV α-DGN). Glycoproteins were enriched using WGA-agarose with 10 mM EDTA. Immunoblotting was performed to detect matriglycan (IIIH11), core α-DG and β-DG (AF6868), and laminin overlay. Molecular weight standards in kilodaltons (kDa) are shown on the left (250, 150, 100, 75, 50, 37, 25, and 20).
