# Supplementary material for: N-terminal domain on dystroglycan enables LARGE1 to extend matriglycan on α-dystroglycan and prevents muscular dystrophy
Source: eLife. 2023 Feb 1;12:e82811. doi: 10.7554/eLife.82811 (PMC9917425; doi:10.7554/eLife.82811)
Supplement: Figure 6—figure supplement 1—source data 1. [file elife-82811-fig6-figsupp1-data1.zip › Figure 6-figure supplement 1-source data 1/Figure 6 - Supp 1_1-18-23_red and green.docx]

**
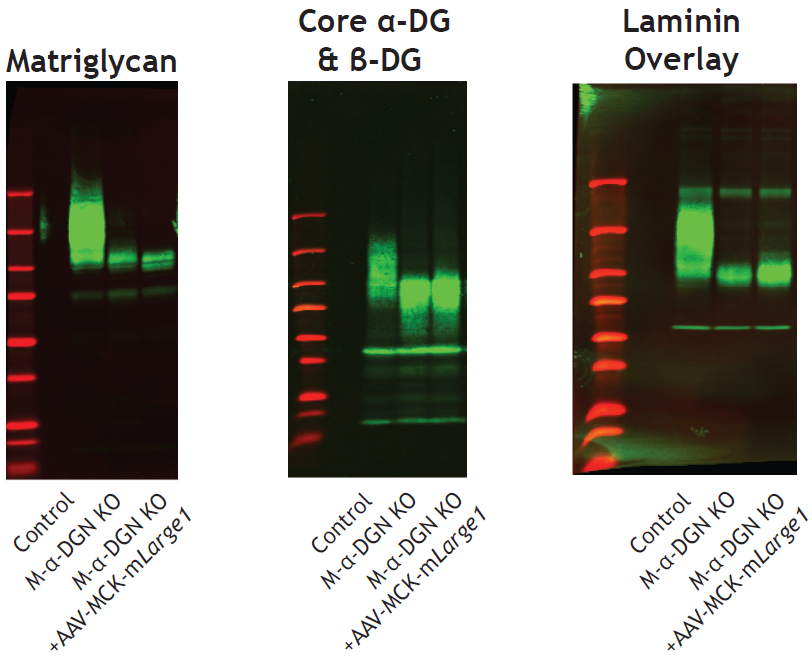
**

**Figure 6-figure supplement 1.** **LARGE1 overexpression does not extend matriglycan on dystroglycan lacking α-DGN**. AAV-MCK-*Large1* was injected into the retro-orbital sinus 10-to-24-week-old M-α-DGN KO mice. Quadriceps skeletal muscle was dissected 10 to 22 weeks after injection from control, M-α-DGN KO, and M-α-DGN KO+AAV-MCK-m*Large1* and used for immunoblotting analysis. Glycoproteins were enriched using WGA-agarose with 10 mM EDTA. Immunoblotting was performed to detect matriglycan (IIIH11), core α-DG and β-DG (AF6868), and laminin (overlay). Molecular weight standards in kilodaltons (kDa) are shown on the left (250, 150, 100, 75, 50, 37, 25, 20, and 15).
