# Supplementary material for: N-terminal domain on dystroglycan enables LARGE1 to extend matriglycan on α-dystroglycan and prevents muscular dystrophy
Source: eLife. 2023 Feb 1;12:e82811. doi: 10.7554/eLife.82811 (PMC9917425; doi:10.7554/eLife.82811)
Supplement: Figure 7—source data 1. [file elife-82811-fig7-data1.zip › Figure 7B-source data 1/Figure 7B_8-22-22_red and green.docx]

**
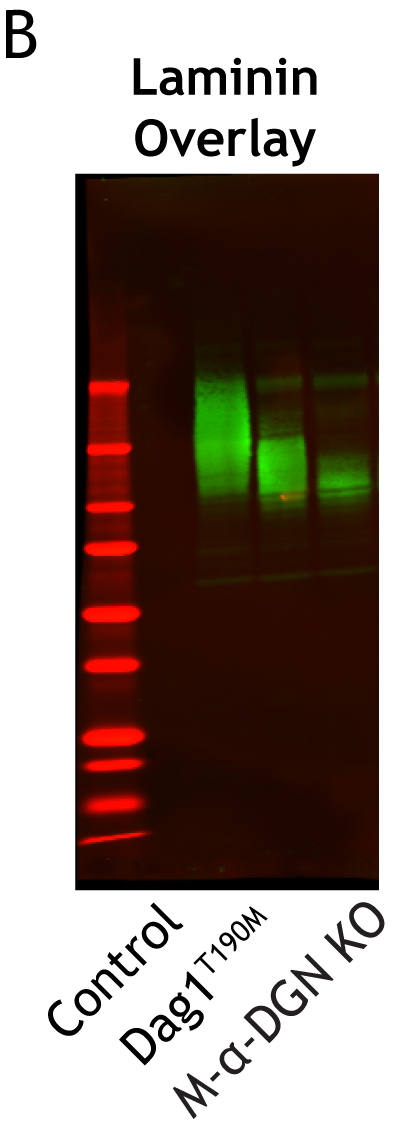
**

**Figure 7.** **Relationship between matriglycan length and dystrophic phenotype. (B)** Immunoblot analysis of quadriceps skeletal muscles from control, *Dag1^T190M^* and M-α-DGN KO mice. Glycoproteins were enriched using WGA-agarose with 10 mM EDTA. Immunoblotting was performed with laminin (laminin overlay). Molecular weight standards in kilodaltons (kDa) are shown on the left (250, 150, 100, 75, 50, 37, 25, 20, 15, and 10).
